# You have ALS: a nurse’s revolt against despair

**DOI:** 10.1177/17449871251407834

**Published:** 2026-01-27

**Authors:** Jenny Aronsen

**Affiliations:** Kristianstad University, Sweden

Friday afternoon, the medical investigation was complete, and I was about to receive the result. The phyician’s voice was close, yet, far away. ‘You have ALS’. I tried to remain professional; after all, I am a nurse. I did not want to cry . . . but I cried. I felt as if the ground beneath me gave way and I fell into bottomless abyss – and fell, and fell.

After a while, I gathered myself together. In some way, I felt a relief. I had known for a very long time, far too long, that something was wrong. People assumed that I had addiction problems, when I lost weight, was exhausted and stumbled. The uncertainty had set my imagination in motion – I had already suffered several imagined serious diseases.

Amyotrophic lateral sclerosis (ALS) is a progressive neurodegenerative disease, that attacks nerve cells controlling voluntary muscles, leading to increased muscle weakness and paralysis. Most patients die because the respiratory muscles become so impaired that the lungs can no longer function. It is a rare, incurable, and fatal disease.

ALS – three simple letters, so much damage.

It can not only take months for someone with ALS to die, but also many years. The most well-known person with ALS, is the scientist Stephen Hawking, who lived with the disease for over 50 years.

During the months/the years one lives with ALS, countless difficult decisions, both large and small, must be made. For example, whether to have a portable ventilator when you can no longer use your lungs to breathe, or whether you consent to CPR if you lose consciousness. These decisions must be made when you are in mentally and relatively physically good condition.

It takes a lifetime to truly know oneself. When I was halfway through life, at 48, I was supposed, not only to learn about myself, but also about my disease – and prepare to die.

We were in the car after the medical appointment, my former husband and I. Silent. Empty. Unable to comfort each other. What were we supposed to do now? Go home to our children and pretend that nothing had happened? Finally, we decided to have one last Taco Friday when everything would seem normal. We would tell them the very next day about my disease. I have struggled with tacos ever since.

How do you tell your children that their mother is going to die? In a society where all information is just a Google search away, we decided to be completely honest. Even though my former husband is a physician and I am a nurse, both with experience of discussing and giving information about serious conditions, it was still unimaginably hard.

Some call ALS the Devil’s disease. It may be so.

Several people with ALS, insist that they ‘'live’' with ALS rather than ‘'suffer’' from it. Some even claim that we are ‘'gifted’' with ALS. Others avoid calling it a ‘'nerve disease’', for fear that it might be confused with mental illnesses – as if that would be worse. Whatever, I simply call it ALS, and it is a bloody disease with no cure.

I have never felt so close to death as during these past 8 years living with ALS, and I have worked as a nurse for many years, with life and death as a part of my daily routines, but of course this was different. Yet, early on, I decided to recover from ALS . . . yes, you may laugh if you want, but I am still here, 8 years later.

The philosopher and author Albert Camus (2018) claimed that, instead of accepting life’s meaninglessness and absurdity, one has to revolt. According to Camus, there is no objective meaning to life – it is up to each individual to fill life with what is meaningful to them. The most important, he argued, was to not accept life’s absurdities without comment.

My way of revolting was to defy the objective truth that ALS was a fatal diagnosis. I did not want to be more dying than anyone else alive. I wanted to carry a realistic hope within me. I would prepare myself and my family for death, but still remain ready to live if a cure was found.

When I told my nurses that I intended to recover from ALS, they pitied me. One male nurse was visibly provoked by the fact that I, as an academically trained nurse, could say something that stupid. Yet, none of them dared to say what they were thinking: ‘'Listen, Jenny, ALS is a disease you will die from . . . with 100% certainty’'.

Their reactions triggered me. It only reflected their own fear of death. I had wished they would talk to me, see me and try to understand my situation. Living with a disease is not only a matter of physical symptoms; it is also how one is met – by misunderstanding and ignorance from others.

According to Sartre (2013), death is a precondition for life. Death forces us to live meaningful and authentic lives. I really do my best.

For my family, the acute mortal fear has calmed down – it is replaced by anticipatory grief. We never talk about death itself, it is our way to cling to life. We all hope for a cure to come. Without hope, you are not able to fully live. Without hope, life becomes only a matter of surviving.

Instead of a bike, I have a wheelchair. Instead of late nights with my friends, I have early evenings with my assistants. That is also a life, my life.
